# Rosmarinic and Sinapic Acids May Increase the Content of Reduced Glutathione in the Lenses of Estrogen-Deficient Rats

**DOI:** 10.3390/nu11040803

**Published:** 2019-04-09

**Authors:** Maria Zych, Weronika Wojnar, Sławomir Dudek, Ilona Kaczmarczyk-Sedlak

**Affiliations:** Department of Pharmacognosy and Phytochemistry, School of Pharmacy with the Division of Laboratory Medicine in Sosnowiec, Medical University of Silesia, Katowice, Jagiellońska 4, 41-200 Sosnowiec, Poland; wwojnar@sum.edu.pl (W.W.); sdudek@sum.edu.pl (S.D.); isedlak@sum.edu.pl (I.K.-S.)

**Keywords:** rosmarinic acid, sinapic acid, lenses, estrogen-deficient rats, oxidative stress, reduced glutathione

## Abstract

Oxidative stress is believed to be associated with both postmenopausal disorders and cataract development. Previously, we have demonstrated that rosmarinic and sinapic acids, which are diet-derived antioxidative phenolic acids, counteracted some disorders induced by estrogen deficiency. Other studies have shown that some phenolic acids may reduce cataract development in various animal models. However, there is no data on the effect of phenolic acids on oxidative stress markers in the lenses of estrogen-deficient rats. The study aimed to investigate whether administration of rosmarinic acid and sinapic acid affects the antioxidative abilities and oxidative damage parameters in the lenses of estrogen-deficient rats. The study was conducted on three-month-old female Wistar rats. The ovariectomized rats were orally treated with rosmarinic acid at doses of 10 and 50 mg/kg or sinapic acid at doses of 5 and 25 mg/kg, for 4 weeks. The content of reduced glutathione (GSH), oxidized glutathione and amyloid β_1-42_, as well as products of protein and lipid oxidation, were assessed. Moreover, the activities of superoxide dismutase, catalase, and some glutathione-related enzymes in the lenses were determined. Rosmarinic and sinapic acids in both doses resulted in an increase in the GSH content and glutathione reductase activity. They also improved parameters connected with protein oxidation. Since GSH plays an important role in maintaining the lens transparency, the increase in GSH content in lenses after the use of rosmarinic and sinapic acids seems to be beneficial. Therefore, both the investigated dietary compounds may be helpful in preventing cataract.

## 1. Introduction

Cataract, a visual impairment characterized by opacification of the lens, may be classified as an age-related disorder. Population-based studies indicate that lens opacities occur more often in women than in men [1,2]. Although estrogen deficiency occurs commonly in elderly women, which might suggest a link between this condition and cataract development, the data on the effect of estrogen on the opacity of the lens is contradictory. On the one hand, the meta-analysis from 2013 [3] showed that hormone replacement therapy reduces the risk of this disease and in vitro studies demonstrated the protective effect of estradiol against oxidative stress in the epithelial cells of the lens [4,5]. On the other hand, although there was a report suggesting that, in the experimental animals exposed to radiation, administration of estradiol may protect against lens opacity [6], other reports showed that treatment with estradiol may induce cataract [7,8]. The role of estrogens in cataract development and cataract dependence on gender has been presented by Zetterberg and Celojevic in a comprehensive review [9].

It is assumed that the development of disorders associated with estrogen deficiency in postmenopausal women, such as vasomotor symptoms, cardiovascular diseases or osteoporosis, is connected with oxidative stress [10,11,12]. Oxidative stress is also considered to be one of the causes of lens opacity [13,14]. Increased production of reactive oxygen species (ROS) and weakened antioxidant system leads to oxidative lens damage, which results in protein aggregation and lens turbidity [14,15]. It is believed that the use of antioxidants in the form of dietary components may be helpful to prevent disorders resulting from post-menopausal oxidative stress [16].

Antioxidants include, among others, phenolic acids, which are components of food products and medicinal plants [17]. The examples of phenolic acids are rosmarinic acid and sinapic acid, which are hydroxycinnamic acid derivatives. Rosmarinic acid is found mainly in plants of the Lamiaceae family, which are widely used as spices and medicinal plants, such as rosemary, spearmint, and lemon balm [18], while sinapic acid occurs in vegetables (especially from the Brassicaceae family, like tronchuda cabbage or broccoli), and fruits (e.g., strawberries or citruses) [19].

Our previous studies showed that both rosmarinic acid and sinapic acid had a positive effect on parameters related to glucose and lipid metabolism, as well as on some parameters of oxidative stress in the serum of ovariectomized rats in the early phase of estrogen deficiency [20,21]. Based on various experimental in vitro and in vivo animal studies, there are also suggestions on the possibility of using phenolic acids, including rosmarinic acid, to reduce cataract development [22,23,24,25]. However, there is still no data on the effects of plant-derived antioxidants, including rosmarinic and sinapic acid on oxidative stress parameters in the lenses exposed to estrogen deficiency. Based on literature data and our previous results, we hypothesized that both rosmarinic acid and sinapic acid may also show a protective antioxidative effect in the lenses of estrogen-deficient rats. Therefore, the study aimed to investigate the effect of rosmarinic and sinapic acids on the antioxidative abilities and oxidative damage parameters in the lenses of ovariectomized rats in the early phase of estrogen deficiency.

## 2. Materials and Methods

### 2.1. Animals and Drugs

The experiment was carried out on three-month-old female Wistar rats. The experiment was conducted under the approval of the Local Ethics Committee in Katowice (permission numbers: 38/2015, 148/2015, and 66/2016). The rats were purchased at the Center of Experimental Medicine, Medical University of Silesia (Katowice, Poland).

In the course of the experiment the following drugs were administered orally to the rats: rosmarinic acid (Sigma-Aldrich, St. Louis, MO, USA), sinapic acid (Sigma-Aldrich, St. Louis, MO, USA) and estradiol hemihydrate (Estrofem, Novo Nordisk A/S, Bagsvard, Denmark). As anesthetics ketamine (Ketamina 10%, Biowet Puławy, Puławy, Poland) and xylazine (Xylapan, Vetoquinol Biowet, Gorzów Wlkp., Poland) were used.

### 2.2. Experimental Design

During the acclimation period (13 days) and during the experiment, the animals had unlimited access to standard feed (Labofeed B, Wytwórnia Pasz “Morawski”, Kcynia, Poland) and drinking water. The rats were divided into 7 groups: (*n* = 10):

- sham-operated control rats (SHAM);
- ovariectomized control rats (OVX);
- ovariectomized rats treated with estradiol at a dose of 0.2 mg/kg (OVX+ESTR);
- ovariectomized rats treated with rosmarinic acid at a dose of 10 mg/kg (OVX+RA10);
- ovariectomized rats treated with rosmarinic acid at a dose of 50 mg/kg (OVX+RA50);
- ovariectomized rats treated with sinapic acid at a dose of 5 mg/kg (OVX+SA5);
- ovariectomized rats treated with sinapic acid at a dose of 25 mg/kg (OVX+SA25).

The OVX+ESTR group of rats was used as a positive control.

As previously described [20,21], rats from the SHAM group underwent a sham surgery, and in the other animals, bilateral ovariectomy was carried out. The sham and ovariectomy surgery were performed under general anesthesia by intraperitoneal (i.p.) administration of the mixture of ketamine and xylazine (87.5 and 12.5 mg/kg i.p., respectively).

Seven days after ovariectomy and sham surgery, the administration of rosmarinic acid, sinapic acid or estradiol to rats started. Phenolic acids and estradiol were administered orally (p.o.) using an intragastric tube once a day for 4 weeks in the form of water solution or suspension, both prepared with the addition of Tween 20 (maximum 1 μL of Tween 20 per 1 mL of water). The sham-operated and ovariectomized control rats were vehicle treated with water containing the same amount of Tween 20, in the same volume of 2 mL/kg p.o. To adjust the volume of administered substances, the rats were weighed twice a week. On the next day after the last administration of drugs and overnight fasting, the animals were sacrificed under general anesthesia (ketamine and xylazine) by cardiac exsanguinations and then the uterus, thymus, liver, right kidney, and eyeballs were removed. Serum obtained from the blood was used to determine biochemical parameters and parameters of oxidative stress, which were previously presented together with body mass and masses of selected organs [20,21]. The lenses were isolated from the eyes, weighed, and homogenized in a glass homogenizer in ice-cold 10 mM phosphate-buffered saline pH 7.4, giving 10% homogenates (*w*/*v*). Part of the total homogenate was frozen, and then used to determine TBARS (thiobarbituric acid reactive substances) and amyloid β_1-42_. The rest was centrifuged at 10,000 × *g* at 4 °C for 15 min. The supernatant was frozen and used to determine the remaining biochemical parameters. All spectrophotometric measurements were carried out with the use of a Tecan Infinite M200 PRO plate reader with Magellan 7.2 software (Tecan Austria, Grödig, Austria).

### 2.3. Determination of Soluble Protein in the Lenses

Determination of soluble protein was conducted according to Lowry’s method [26]. BSA was used to prepare the calibration curve, and the protein content was expressed in milligram per gram of the lens.

### 2.4. Determination of Superoxide Dismutase and Catalase Activities and Oxidative Damage Products Content in the Lenses

To determine the activities of the following antioxidant enzymes: superoxide dismutase (SOD) and catalase (CAT), Cayman kits (Cayman Chemical MI, USA) were used. The activities of SOD and CAT were expressed in U or nanomole/min, respectively, per milligram of protein.

The method of Ohkawa et al. [27] was used to determine the content of TBARS (thiobarbituric acid reactive substances) in the total homogenate of the lenses. This method is based on the reaction between lipid peroxidation products and thiobarbituric acid. TBARS content is expressed in nanomole per gram of the lens. The intensity of the obtained color was determined spectrophotometrically at the wavelength of 535 nm. To establish a standard curve, 1,1,3,3-tetraethoxypropane (Sigma-Aldrich, St. Louis, MO, USA) was used.

The concentration of advanced oxidation protein products (AOPP) in the lens homogenate was determined using spectrophotometric method described by Witko-Sarsat et al. [28]. The calibration curve was established using chloramine T (Sigma-Aldrich, St. Louis, MO, USA), while the absorbance was measured at the wavelength of 340 nm. The content of AOPP was expressed in nanomole chloramine T equivalents per milligram of protein.

### 2.5. Determination of Glutathione-related Enzymes Activities in the Lenses

Glutathione peroxidase (GPx) and glutathione reductase (GR) activities were determined using Cayman kits. The activities of GPx and GR were expressed in nanomole of reduced nicotinamide adenine dinucleotide phosphate (NADPH) oxidized during 1 min per milligram of protein.

Activity of glucose-6-phosphate dehydrogenase (G6PD) was measured with Pointe Sci. Kit (Pointe Scientific, Canton, MI, USA), while to determine the activity of γ-glutamyl transpeptidase (GGT), the BioSystems kit was used (Costa Brava, Barcelona, Spain). The activity of G6PD was expressed in nanomole of NADP^+^ reduced during 1 min per milligram of protein and the activity of GGT was expressed in nanomole of 3-carboxy-4-nitroaniline formed during 1 min per milligram of protein.

### 2.6. Determination of Glutathione in the Lenses

The concentration of total glutathione (TotGSH) and the concentration of oxidized glutathione (GSSG) in the lens homogenate was determined by Cayman kit (Cayman Chemical MI, USA). The concentration of reduced glutathione (GSH) was calculated according to the formula: GSH = TotGSH - 2×GSSG (nmol/mL), and then the GSH/GSSG ratio was determined. The content of GSH and GSSG in the lenses is expressed in nanomole per milligram of protein.

### 2.7. Determination of Amyloid β_1-42_ Content in the Lenses

ELISA kit (Bioassay Technology Laboratory, Shanghai, Yangpu, China) was used to determine the content of amyloid β_1-42_. Following the manufacturer’s instructions, total homogenates were centrifuged at 2500 RPM for 20 min, and amyloid β_1-42_ was determined in the obtained supernatants. The content of amyloid β_1-42_ was expressed in nanogram per gram of the lens.

### 2.8. Statistical Analysis

The results are presented as the arithmetic mean ± SEM. One-way ANOVA followed by Duncan’s post-hoc test were applied to assess statistical significance of the results (Statistica 12 software, StatSoft Polska, Kraków, Poland). The results were assumed statistically significant if *p* ≤ 0.05.

## 3. Results

### 3.1. Effect of Rosmarinic Acid and Sinapic Acid on the Lens Mass and Lens Soluble Protein Content

The average mass of the lens, as well as the soluble protein content in the lenses of the ovariectomized control rats, did not change statistically as compared to the lenses in the sham-operated rats. The administration of rosmarinic acid or sinapic acid in both doses did not lead to any changes in the average mass of the lens or in the content of soluble protein of the lenses compared to ovariectomized control rats. Similarly, administration of estradiol to the ovariectomized rats did not cause any changes in these parameters (Table 1).

### 3.2. Effect of Rosmarinic Acid and Sinapic Acid on Superoxide Dismutase and Catalase Activities and on Oxidative Damage Products Content in the Lenses

**Figure 1:**
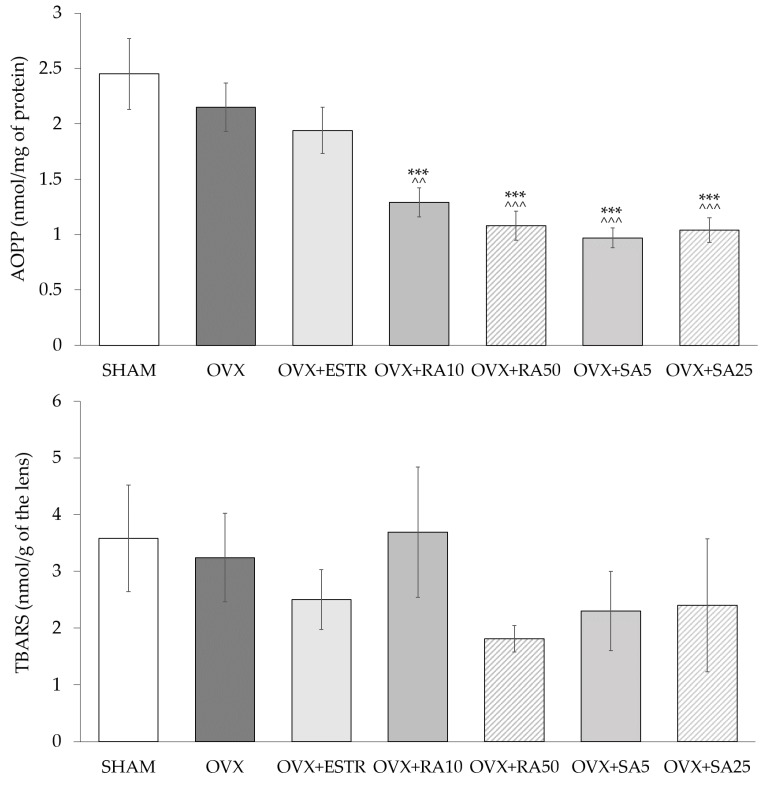
Effect of rosmarinic acid and sinapic acid on the AOPP and TBARS content in the lenses of ovariectomized rats. Rosmarinic acid at doses of 10 mg/kg (OVX + RA10) and 50 mg/kg (OVX + RA50), sinapic acid at doses of 5 mg/kg (OVX + SA5) and 25 mg/kg (OVX + SA25) or estradiol at a dose 0.2 mg/kg (OVX + ESTR) were administered orally to ovariectomized rats, once daily for 28 days. SHAM: sham-operated control rats; OVX: ovariectomized control rats; TBARS: thiobarbituric acid reactive substances; AOPP: advanced oxidation protein products. Results are presented as the mean ± SEM. One-way ANOVA followed by Duncan’s test were used for evaluation of the significance of the results. *** *p* < 0.001: significant differences with regard to the SHAM control rats. ^^ *p* < 0.01, ^^^ *p* < 0.001—significant differences with regard to the OVX control rats. No statistically significant differences in results for TBARS were demonstrated by ANOVA.

In the lenses of the ovariectomized rats, no significant changes in the SOD and CAT activities were observed compared to the sham-operated rats. The administration of estradiol and phenolic acids did not cause any significant changes in the activities of these enzymes when compared to the ovariectomized control rats (Table 2). Estrogen deficiency in the ovariectomized rats did not affect the content of AOPP and TBARS in the lenses as compared to the sham-operated rats. The use of rosmarinic acid at doses of 10 and 50 mg/kg and sinapic acid at doses of 5 and 25 mg/kg p.o. led to a decrease of the AOPP content in the lenses in comparison to the ovariectomized control rats, whereas estradiol did not exert such an effect. The administration of estradiol and phenolic acids did not significantly change the content of TBARS in the lenses as compared to the ovariectomized rats (Figure 1).

### 3.3. Effect of Rosmarinic Acid and Sinapic Acid on Glutathione-Related Enzymes Activities in the Lenses

The GR, G6PD, and GGT activities in the lenses were decreased in the ovariectomized control rats in a statistically significant manner, whereas the GPx activity showed no statistically significant difference in comparison to the sham-operated control rats. The administration of estradiol to the estrogen-deficient rats did not affect the activities of the examined glutathione-related enzymes. A statistically significant increase in the GR activity was observed after the administration of rosmarinic acid at doses of 10 and 50 mg/kg and sinapic acid at doses of 5 and 25 mg/kg. After administration rosmarinic acid at 50 mg/kg, there was a tendency to increase (*p* = 0.058) in the G6PD activity, whereas the administration of 25 mg/kg of sinapic acid significantly increased the activity of this enzyme when compared to the ovariectomized control rats. Rosmarinic acid and sinapic acid had no effect on the activities of GPx and GGT (Table 3).

### 3.4. Effect of Rosmarinic Acid and Sinapic Acid on Glutathione Content in the Lenses

**Figure 2:**
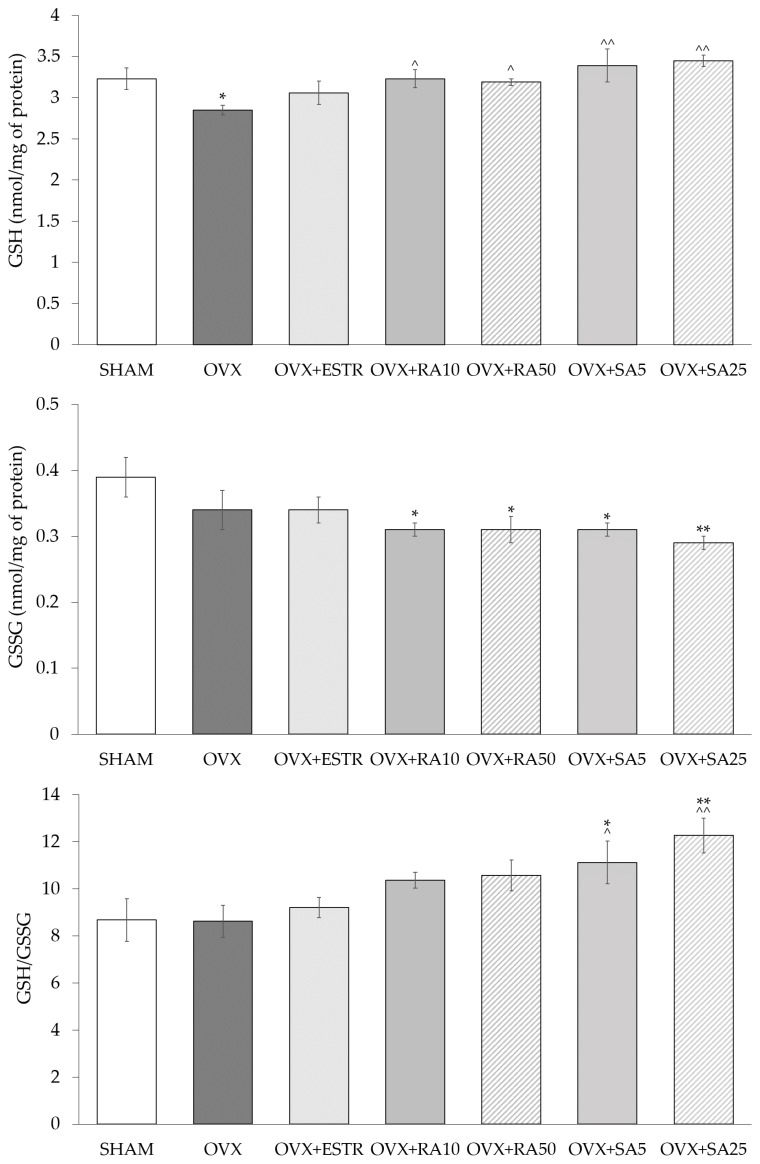
Effect of rosmarinic acid and sinapic acid on the GSH and GSSG content and on the GSH/GSSG ratio in the lenses of ovariectomized rats. Rosmarinic acid at doses of 10 mg/kg (OVX + RA10) and 50 mg/kg (OVX + RA50), sinapic acid at doses of 5 mg/kg (OVX + SA5) and 25 mg/kg (OVX + SA25) or estradiol at a dose 0.2 mg/kg (OVX+ESTR) were administered orally to ovariectomized rats, once daily for 28 days. SHAM: sham-operated control rats; OVX: ovariectomized control rats; GSH: reduced glutathione; GSSG: oxidized glutathione. Results are presented as the mean ± SEM. One-way ANOVA followed by Duncan’s test were used for evaluation of the significance of the results. * *p* ≤ 0.05, ** *p* < 0.01: significant differences with regard to the SHAM control rats. ^ *p* ≤ 0.05, ^^ *p* < 0.01: significant differences with regard to the OVX control rats.

A statistically significant decrease in the content of the reduced glutathione (GSH) in the lenses was observed while the content of the oxidized glutathione (GSSG) and the GSH/GSSG ratio did not change in the ovariectomized control rats compared to the sham-operated rats. The administration of estradiol did not change the content of GSH and GSSG or GSH/GSSG ratio, whereas the administration of phenolic acids (rosmarinic acid at 10 and 50 mg/kg and sinapic acid at dose 5 and 25 mg/kg) resulted in the statistically significant increase in the GSH content in the lenses, without impact on the GSSG content when compared to the ovariectomized control rats. The use of rosmarinic acid in the estrogen-deficient rats at both doses did not affect the GSH/GSSG ratio, while the use of sinapic acid at both doses caused a significant increase in the GSH/GSSG ratio, compared to the ovariectomized control rats (Figure 2).

### 3.5. Effect of Rosmarinic Acid and Sinapic Acid on Amyloid β_1-42_ Content in the Lenses

**Figure 3:**
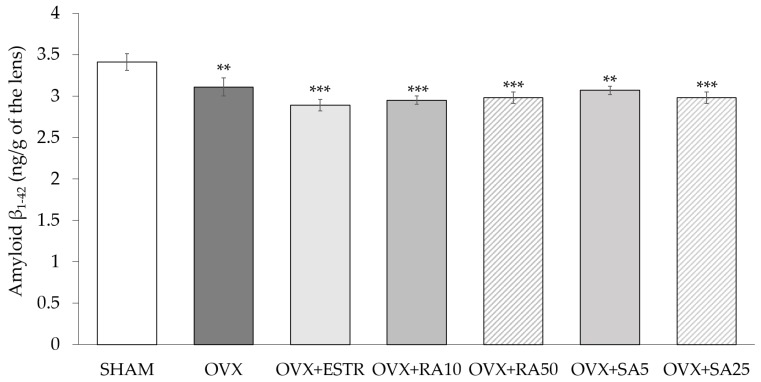
Effect of rosmarinic acid and sinapic acid on the amyloid β_1-42_ content and on the GSH/GSSG ratio in the lenses of ovariectomized rats. Rosmarinic acid at doses of 10 mg/kg (OVX + RA10) and 50 mg/kg (OVX + RA50), sinapic acid at doses of 5 mg/kg (OVX + SA5) and 25 mg/kg (OVX + SA25) or estradiol at a dose 0.2 mg/kg (OVX+ESTR) were administered orally to ovariectomized rats, once daily for 28 days. SHAM: sham-operated control rats; OVX: ovariectomized control rats. Results are presented as the mean ± SEM. One-way ANOVA followed by Duncan’s test were used for evaluation of the significance of the results. ** *p* < 0.01, *** *p* < 0.001: significant differences with regard to the SHAM control rats.

The content of amyloid β_1-42_ in the lenses of the ovariectomized rats significantly decreased as compared to the sham-operated rats. The administration of rosmarinic acid and sinapic acid did not result in any statistically significant changes in the content of amyloid β_1-42_. Likewise, treatment with estradiol did not affect the amyloid β_1-42_ content in the lenses of ovariectomized rats (Figure 3).

## 4. Discussion

The lens is a transparent structure located in the front part of the eye. It is the most important part of the optical system of the eye, which projects a reduced, inverted, and exceptionally clear image on the retina. The lack of cell nuclei and other light-scattering organelles contributes to the transparency of the lens. Light scattering is also minimized due to the close apposition of the lens fiber cells [29]. It has recently been pointed out that the lens is not a passive optical component, but an active tissue (which may, for example, protect the anterior segment of the eye from oxygen or its metabolites, as well as can release GSH and adenosine triphosphate (ATP) to other eye tissues), the removal of which can contribute to the development of other eye diseases [30]. The artificial lens is not capable of performing metabolic functions but only serves as an optical element. Therefore, although contemporary cataract surgery is safe, it is still recommended to avoid removing lenses and to put more emphasis on preventing cataract formation.

Scientific reports based on observational studies indicate that a well-balanced diet rich in vegetables and fruits, containing about 150 g of protein, high intake of vitamin C, vitamin E, and a reduced amount of simple sugars, as well as supplementation with other vitamins or carotenoids, may contribute to delaying the cataract progression [31]. According to the in vitro and in vivo experiments, dietary components derived from medicinal plants, such as flavonoids, phenolic acids, terpenes, carotenoids or phytosterols, seem to be effective in preventing opacity of the lenses [22,23,31]. They can act through various mechanisms, of which the most important is anti-oxidative and anti-glycating activities [23].

The aim of the presented study was to investigate the effect of dietary components: rosmarinic acid and sinapic acid on antioxidative abilities parameters (GSH and enzymes associated with GSH and SOD, CAT), as well as products of oxidative damage from lipids and proteins (TBARS and AOPP, respectively) in the lenses obtained from the rats 5 weeks after ovariectomy. Rosmarinic acid was administered to animals at doses of 10 and 50 mg/kg, while sinapic acid at doses of 5 and 25 mg/kg. As we discussed before [20,21] the doses of phenolic acids have been selected so that the smaller ones (10 mg/kg rosmarinic acid and 5 mg/kg sinapic acid) correspond to the amount that can be consumed in the diet. Five times higher doses were used to determine whether they exert a stronger therapeutic effect than achievable dietary doses. The doses used in this experiment could be considered safe as acute toxicity tests conducted for rosmarinic acid and sinapic acid revealed that both the acids are non-toxic even at a dose of 2000 mg/kg when administered orally to rats [32,33].

Estrogen deficiency in ovariectomized animals was manifested by a decrease in estradiol and progesterone concentration in the serum, a decrease in uterus mass, and enhanced body mass gain, as well as the changes in parameters related to glucose and lipid metabolism [20,21]. We have previously reported that the use of estradiol, sinapic acid [21], and rosmarinic acid [20] in these rats had a positive effect on the serum parameters associated with glucose and lipid metabolism and also increased serum GSH concentration.

The increase in GSH content in the lenses, which was reduced by estrogen deficiency, was also observed in the present study in estrogen-deficient rats administered with rosmarinic acid and sinapic acid. GSH plays an important role in maintaining the lens transparency, and simultaneously in the regulation of the lens redox state [34,35,36]. GSH may form reversible disulfide bonds with protein thiol groups. Therefore, it protects proteins from permanent oxidation and, in result, from their aggregation and loss of function [37]. Moreover, GSH is a cofactor for numerous enzymes, such as thioltransferase (TT-ase), which uses GSH to dethiolate protein-thiol disulfides [36,38]. GSH can also be used by other antioxidative enzymes, such as glutathione peroxidase (GPx), to neutralize H_2_O_2_ [36] or glutathione S transferases (GST). GSTs of many classes (such as pi or mu) use GSH as a substrate to neutralize electrophilic xenobiotics [39], regulate pro- and antiapoptotic pathways in many tissues [40,41,42] and polymorphism in genes encoding GSTs may be an important risk factor in cataractogenesis [43,44,45]. GSH synthesis takes place in the lens epithelium and the outer part of the lens cortex. The required amino acids are supplied from the aqueous humor and from the decomposition of GSH in the gamma-glutamyl cycle in which the γ-glutamyl transferase (GGT) plays a major role. The role of GGT is to break down extracellular GSH, GSSG, and S-glutathione conjugates, thus, providing cells with amino acids which are necessary for intracellular GSH synthesis [34]. GSH, as a complete tripeptide, may also be transferred to the lens from aqueous humor. The content of GSH in the lenses decreases with age. It is believed that this is a result of, for example, a decreased glutamate cysteine ligase (GCL) activity, and, hence, a reduction in the GSH de novo synthesis, but also weakening of the GSH regeneration system from the oxidized form, which includes GR and G6PD [34].

In this study, the reduced content of GSH in the lenses of the ovariectomized control rats compared to the lenses of the sham-operated rats was observed simultaneously with decreased GR, G6PD, and GGT activities. GR is necessary to reduce GSSG using NADPH, while G6PD catalyzes the first phase of the pentose phosphate pathway, during which glucose-6 phosphate is transformed into 6-phosphoglucono-δ lactone, and NADP^+^ is reduced to NADPH [34,46]. NADPH is essential for the activity of many enzymes, including GR. Lowering of the GSH content may, therefore, be a result of a weakened regeneration from GSSG, but there is also a possibility that the decrease of GSH content may be an effect of the reduced synthesis resulting from decreased GCL activity. Based on the results obtained in this study, the determination of the mechanisms responsible for lowering the GSH content in estrogen-deficient rats is not entirely possible. It seems to be quite surprising that even though GR, G6PD, and GGT activities are lowered in the lenses of the rats which underwent ovariectomy, the GSSG content is not elevated and GSH/GSSG ratio remained unchanged when compared to the sham-operated animals. In the report of Umapathy et al. [47], the authors suggest that the excess of GSSG is exported from the lens to the neighboring structures as an early response to oxidative stress to minimize the possible damage and maintain lenticular GSH redox state [47]. A decreased GSH content in the lenses is well documented in various rat cataract models [48,49,50,51,52]. However, there are only few reports describing the GSH content or antioxidative abilities in the lenses of the laboratory animals with estrogen deficiency [53,54]. A study conducted on ovariectomized mice showed that GSH content in the lenses did not change when compared with control animals [53]. Acer et al. [54] examined total non-enzymatic antioxidant content in the lenses of ovariectomized rats conducting total antioxidant capacity (TAC) test and noted that TAC in the lenses of estrogen-deficient rats was significantly lower than in the lenses of control rats [54]. The results for GSH content in the lenses obtained in our study overlap with these for TAC presented by Acer et al. [54], possibly due to the fact that GSH is a predominant non-enzymatic antioxidant in the lenses [47].

The increase in the GSH content in the lenses of ovariectomized rats treated with rosmarinic acid and sinapic acid in both doses was accompanied by an increase in the GR activity. There was also a tendency to increase and significant increase of G6PG activity after the use of 50 mg/kg rosmarinic acid and 25 mg/kg sinapic acid, respectively. No effect on GPx and GGT activities was noted after administration of both phenolic acids regardless of the used dose. Therefore, it seems that the increase in the GSH content in the lenses after the use of phenolic acids may partly result from increased regeneration from GSSG. It is also probable that the increase in the GSH content is due to its increased synthesis, as rosmarinic acid is described to up-regulate the catalytic subunits of GCL in hepatic stellate cells [55]. There are reports indicating that the use rosmarinic acid [56,57] and sinapic acid [58], in different experimental rodent models, led to the increased content of GSH in various tissues and organs, such as kidneys and liver [56,57,58]. An important indicator of cellular redox status as well as for the redox state in tissues is the GSH/GSSG ratio [59]. In the present study, there was a statistically significant increase in this ratio in the lenses of the estrogen-deficient rats after treatment with both doses of sinapic acid.

A period of 35 days after ovariectomy in rats corresponds to approximately 3.3 years in postmenopausal women [60]. In the early postmenopausal period, some changes in the organism are not very pronounced. This may explain why in the present study there were no statistically significant changes in the activities of antioxidant enzymes or the content of oxidative damage parameters, such as TBARS or AOPP. Estradiol and phenolic acids in the present study did not affect the activities of SOD, CAT, and the content of TBARS, but both the rosmarinic acid and sinapic acid reduced the AOPP content in the lenses. Although the content of AOPP did not increase in ovariectomized rats as compared to the sham-operated rats, the reduction in the AOPP content seems to be a favorable change, since AOPP may promote ROS formation via the receptor for advanced glycation end products (RAGE)-dependent pathway [61]. Reduction in the AOPP content was also observed as a result of using the plant-derived antioxidants, such as diosmin, naringenin or resveratrol, in the lenses of rats with experimentally induced diabetes [62,63,64].

One of the parameters which depict changes occurring in the lens during cataract development is the content of amyloid β_1-42_. In rats, 5 weeks after performing ovariectomy, a reduction in the amyloid β_1-42_ content was observed, which is consistent with the results indicating that its expression is reduced in the early and middle stages of age-related cataract development in human lens epithelial cells [65]. In addition, in Upjohn Pharmaceutical Limited (UPL) rats (a dominant hereditary cataract model derived from Sprague-Dawley rats), there was no increase in the content of amyloid β_1-42_ in the lenses until complete opacity occurred [66]. There was no effect of rosmarinic acid and sinapic acid on the amyloid β_1-42_ content in the lenses.

In our previous study, we observed that even though estradiol administered orally to ovariectomized rats caused an increase in the uterus mass and a decrease in the thymus mass (estrogenic activity), it did not increase the estradiol level in the serum [21]. In the presented study it was noted that oral administration of estradiol did not affect oxidative stress-related parameters in the lenses of ovariectomized rats, including the GSH content. Similar findings were made in another study, in which estradiol administered to estrogen-deficient rats revealed no effect on apoptosis rate in the lens epithelial cells, which was increased in the ovariectomized control rats [67]. Unlike the treatment with estradiol, administration of sinapic acid at both doses caused a significant increase in the estradiol concentration in the serum of the ovariectomized rats [21]. What is more, after treatment with rosmarinic acid at the higher dose (50 mg/kg) there was a trend to an increase in the estradiol concentration in the serum of ovariectomized rats [20]. Therefore, although, it appears that the action of rosmarinic and sinapic acids on GSH content in the lenses of ovariectomized rats results rather from their anti-oxidative activity, there is also a possibility that the mechanism of their action could be somehow estrogen-dependent.

Our research has some limitations. First of all, further studies are required to determine unequivocally whether mechanism underlying changes in GSH content in the lenses after administration of the phenolic acids is connected with direct effects of these compounds on anti-oxidative status or rather with their phytoestrogenic activity. Moreover, the present study did not assess the effect of rosmarinic acid and sinapic acid on the cytoplasmatic expression of antioxidant enzymes. To affirm our results, further molecular studies using Western blot or real-time PCR could be helpful. Since there is a possibility of simultaneous consumption of rosmarinic acid and sinapic acid, it would also be interesting to investigate the effects of these phenolic acids combined.

## 5. Conclusions

Rosmarinic and sinapic acids contributed to the increase in GSH content in the lenses of rats in the early phase of estrogen deficiency. Due to the important role of GSH in maintaining the transparency of the lenses, it seems that these phenolic acids may exert a beneficial effect on the redox status in the eye lenses of ovariectomized rats, and, thus, may be a supporting factors in the prevention of cataract formation.

## Figures and Tables

**Table 1:** Effects of rosmarinic acid and sinapic acid on the average lens mass and lens soluble protein content in ovariectomized rats.

Parameter/Group	SHAM	OVX	OVX + ESTR	OVX + RA10	OVX + RA50	OVX + SA5	OVX + SA25
Average mass of the lens (g)	0.059 ± 0.002	0.058 ± 0.003	0.055 ± 0.002	0.055 ± 0.001	0.056 ± 0.002	0.056 ± 0.002	0.055 ± 0.001
Soluble protein (mg/g of the lens)	280.6 ± 6.8	302.5 ± 7.8	295.8 ± 5.8	290.4 ± 3.2	291.7 ± 7.2	291.9 ± 5.2	286.7 ± 4.2

Rosmarinic acid at doses of 10 mg/kg (OVX+RA10) and 50 mg/kg (OVX+RA50), sinapic acid at doses of 5 mg/kg (OVX+SA5) and 25 mg/kg (OVX+SA25) or estradiol at a dose 0.2 mg/kg (OVX+ESTR) were administered orally to ovariectomized rats, once daily for 28 days. SHAM: sham-operated control rats; OVX: ovariectomized control rats. Results are presented as the mean ± SEM. No statistically significant differences in results for both parameters were demonstrated by ANOVA.

**Table 2:** Effect of rosmarinic acid and sinapic acid on the superoxide dismutase (SOD) and catalase (CAT) activities in the lenses of ovariectomized rats.

Parameter/Group	SHAM	OVX	OVX + ESTR	OVX + RA10	OVX + RA50	OVX + SA5	OVX + SA25
SOD (U/mg of protein)	0.194 ± 0.017	0.156 ± 0.003	0.170 ± 0.007	0.164 ± 0.005	0.174 ± 0.006	0.171 ± 0.012	0.167 ± 0.002
CAT (nmol/min/mg of protein)	0.085 ± 0.012	0.033 ± 0.009	0.052 ± 0.014	0.075 ± 0.014	0.076 ± 0.020	0.060 ± 0.018	0.063 ± 0.010

Rosmarinic acid at doses of 10 mg/kg (OVX + RA10) and 50 mg/kg (OVX + RA50), sinapic acid at doses of 5 mg/kg (OVX + SA5) and 25 mg/kg (OVX + SA25) or estradiol at a dose 0.2 mg/kg (OVX + ESTR) were administered orally to ovariectomized rats, once daily for 28 days. SHAM: sham-operated control rats; OVX: ovariectomized control rats; SOD: superoxide dismutase (1 U of SOD determines the amount of enzyme required to exhibit 50% dismutation of the superoxide radical); CAT: catalase. Results are presented as the mean ± SEM. No statistically significant differences in results for SOD and CAT were demonstrated by ANOVA.

**Table 3:** Effects of rosmarinic acid and sinapic acid on the glutathione-related enzymes activities in the lenses of ovariectomized rats.

Parameter/Group	SHAM	OVX	OVX + ESTR	OVX + RA10	OVX + RA50	OVX + SA5	OVX + SA25
GPx (nmol/min/mg of protein)	2.31 ± 0.08	2.15 ± 0.08	2.04 ± 0.07	2.26 ± 0.11	2.21 ± 0.06	2.24 ± 0.08	2.28 ± 0.05
GR (nmol/min/mg of protein)	0.367 ± 0.047	0.220 ± 0.036 **	0.291 ± 0.034	0.357 ± 0.027 ^^	0.352 ± 0.026 ^^	0.323 ± 0.026 ^	0.346 ± 0.021 ^
G6PD (nmol/min/mg of protein)	1.99 ± 0.10	1.30 ± 0.14*	1.39 ± 0.17 *	1.52 ± 0.19	1.83 ± 0.16	1.47 ± 0.13	2.00 ± 0.27 ^
GGT (nmol/min/mg of protein)	0.039 ± 0.005	0.024 ± 0.004*	0.028 ± 0.003	0.024 ± 0.004 *	0.020 ± 0.002 **	0.025 ± 0.005 *	0.031 ± 0.002

Rosmarinic acid at doses of 10 mg/kg (OVX + RA10) and 50 mg/kg (OVX + RA50), sinapic acid at doses of 5 mg/kg (OVX + SA5) and 25 mg/kg (OVX + SA25) or estradiol at a dose 0.2 mg/kg (OVX + ESTR) were administered orally to ovariectomized rats, once daily for 28 days. SHAM: sham-operated control rats; OVX: ovariectomized control rats; GPx: glutathione peroxidase; GR: glutathione reductase, G6PD: glucose-6-phosphate dehydrogenase. Results are presented as the mean ± SEM. One-way ANOVA followed by Duncan’s test was used for evaluation of the significance of the results. * *p* ≤ 0.05, ** *p* < 0.01: significant differences with regard to the SHAM control rats. ^ *p* ≤ 0.05, ^^ *p* < 0.01: significant differences with regard to the OVX control rats. No statistically significant differences in results for GPx were demonstrated by ANOVA.
